# Cerebral oxygenation during induction of therapeutic hypothermia after cardiac arrest

**DOI:** 10.1186/cc10890

**Published:** 2012-03-20

**Authors:** I Meex, J Dens, F Jans, C De Deyne

**Affiliations:** 1 Ziekenhuis Oost-Limburg, Genk, Belgium

## Introduction

Induced mild hypothermia (32 to 34°C) improves survival and neurological outcome after CA. Near-infrared spectroscopy (NIRS) measures cerebral tissue oxygen saturation (SctO_2_). As of today, no data are available on SctO_2 _monitoring during therapeutic hypothermia (TH). Therefore, SctO_2 _was measured in this study during the first 36 hours after CA.

## Methods

After IRB approval, data were collected from 23 patients. Cold saline (30 ml/kg) was administered as soon as possible after hospital admission. TH (33°C) was induced by endovascular or surface cooling and maintained for 24 hours. All patients were sedated (propofol/remifentanil) for the duration of TH. NIRS sensors were bilaterally applied to the frontotemporal area before start of TH. Patients were monitored during induction, maintenance and recovery of TH.

## Results

Of 23 patients, 11 patients did not survive until hospital discharge due to post-ischemic brain damage. Twelve patients survived until hospital discharge, of whom eight without any neurological impairment. Temperature at admission was 34.6°C (±0.5°C). Patients reached the target temperature of 33°C, 4 hours after induction of TH. Two patients died during maintenance of TH due to refractory hemodynamic shock. In all patients, SctO_2 _values started above 65%. Two and a half hours after induction of TH, SctO_2 _values decreased with 9% (±3%). The decrease in cerebral oxygenation during induction of TH was not associated with a major change in hemodynamic parameters (MAP before induction of TH: 79 mmHg ± 19; at 33°C: 82 mmHg ± 9), nor with a major change in systemic oxygenation (SpO_2 _before TH: 99% ± 1; at 33°C: 97% ± 3). In patients who survived until hospital discharge, SctO_2 _returned to baseline values, 3.5 hours after induction of TH, before the target temperature of 33°C was reached. In patients who did not survive the hospital stay, SctO_2 _remained lower than baseline values until the target temperature was reached. In these nonsurvivors, SctO_2 _values did only return to baseline values during maintenance of TH (10 hours after induction of TH). During maintenance of TH and rewarming (0.3°C), no further significant changes in SctO_2 _values were observed.

## Conclusion

Noninvasive monitoring revealed a decrease in cerebral oxygenation during induction of mild hypothermia in patients after cardiac arrest. We observed a difference in oxygenation between hospital survivors and nonsurvivors.

